# Commercially available garden products as important sources of antibiotic resistance genes—a survey

**DOI:** 10.1007/s11356-021-13333-7

**Published:** 2021-04-09

**Authors:** Marisol Cira, Cristina M. Echeverria-Palencia, Ileana Callejas, Karina Jimenez, Rafael Herrera, Wei-Cheng Hung, Nicolas Colima, Amanda Schmidt, Jennifer A. Jay

**Affiliations:** 1 Civil and Environmental Engineering Department, University of California Los Angeles, 420 Westwood Plaza, Los Angeles, CA 90095 USA; 2 Institute of the Environment and Sustainability, University of California Los Angeles, LaKretz Building, Los Angeles, CA 90095 USA

**Keywords:** Source of antibiotic resistance, Antibiotic resistance genes, Garden products, Recently landscaped soils, Native soils, qPCR

## Abstract

The dissemination of antibiotic resistance genes (ARGs) in the environment contributes to the global rise in antibiotic resistant infections. Therefore, it is of importance to further research the exposure pathways of these emerging contaminants to humans. This study explores commercially available garden products containing animal manure as a source of ARGs in a survey of 34 garden products, 3 recently landscaped soils, and 5 native soils. DNA was extracted from these soils and quantified for 5 ARGs, *int*I1, and 16S rRNA. This study found that both absolute and relative gene abundances in garden products ranged from approximately two to greater than four orders of magnitude higher than those observed in native soils. Garden products with *Organic Materials Review Institute (*OMRI) certification did not have significantly different ARG abundances. Results here indicate that garden products are important sources of ARGs to gardens, lawns, and parks.

**Supplementary Information:**

The online version contains supplementary material available at 10.1007/s11356-021-13333-7.

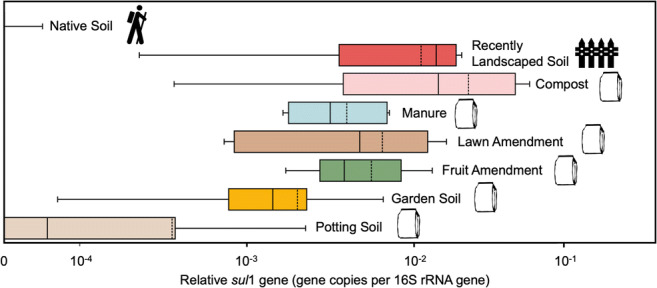

## Introduction

Antibiotic resistance threatens the global response to infectious disease, with over 700,000 deaths in 2014 projected to increase to 10 million by 2050 (O’Neill 2014). Concurrently, over 11 million kg of antibiotics are administered to livestock annually for growth promotion and disease prevention (USFDA 2018). An estimated 30–90% of antibiotics administered for agricultural use are not metabolized prior to excretion and are instead introduced into the environment where they exert a selective pressure for antimicrobial resistance (Sarmah et al. 2006). Additionally, consistent animal exposure to antibiotics drives selection within livestock gut microbiomes, resulting in the excretion of antibiotic-resistant bacteria (ARB) into the environment (Looft et al. 2012). Confined animal feeding operations (CAFOs) have widely been confirmed as sources of anthropogenic antimicrobial resistance into the environment (Heuer et al. 2011a; McEachran et al. 2012; Sancheza et al. 2016) due to use of antibiotics in animal feed. While antibiotic resistance genes (ARGs) also exist in native soils due to selective pressures that occur naturally in the environment (Allen et al. 2010; Hall and Barlow 2004), archived agricultural soils show a significant increase in ARG abundance since 1940, corresponding with the increased use of antibiotics in animal husbandry (Knapp et al. 2010).

Manure from CAFOs is also frequently mixed with soil in rural and urban settings to restore and replenish nutrients. This process constitutes a significant pathway of ARG dissemination from agriculture, with a substantial body of literature confirming that such repeated manure application results in propagation of both antibiotics and ARGs in fields (Dungan et al. 2019; Fahrenfeld et al. 2014; Heuer et al. 2011b; Jechalke et al. 2013; Marti et al. 2013; Sandberg and LaPara 2016).

However, there is an important knowledge gap with regard to commercially available garden products containing animal manure, which are readily obtainable by the general public. Animal manure contains antibiotic-resistant bacteria (Fahrenfeld et al. 2014; Liu et al. 2016; Marti et al. 2013; Wang et al. 2015), representing a potential route of consumer exposure to ARGs via fertilized gardens, lawns, and parks. In addition to a lack of data on ARG content, there is a lack of regulation over garden product branding. For example, for food, fiber, and feed products, the term “organic” is regulated by the Code of Federal Regulations (Code of Federal Regulations n.d.), but for fertilizers, the term “organic” is not federally regulated. Instead the Organic Materials Review Institute (OMRI) and the Demeter Association certifications verify that fertilizers meet the USDA National Organic Program (NOP) regulations. However, the NOP regulations refer to any organic matter containing plant and animal material, including raw manure, as “organic.” In addition, there is little transparency on fertilizer treatment prior to purchase, with garden product packaging and online descriptions lacking this information and with the NOP regulations only explicitly outlining treatment methods for vermicomposting. Further, unlike the Code of Federal Regulations, the NOP regulations do not consider propagation of antibiotic resistance, either through regulating antibiotic, ARB, or ARG content (National Organic Program Handbook n.d.).

It is critical to comprehensively consider environments existing at the nexus of human-ARG interaction. Understanding sources and scope of dissemination will prove critical in determining potential points of mitigation as well as approach. Therefore, in this study, we sought to measure ARG content in 34 commercially available garden products intended for home use, 3 recently landscaped soils from a community garden, residential lawn, and a park, and 5 native soils from hiking trails. Two sulfonamide resistance genes, *sul*1 and *sul*2, two tetracycline resistance genes, *tet*(L) and *tet*(W), one macrolide resistance gene, *erm*(F), one class 1 integron-integrase gene, *intI*1, and a total bacterial surrogate, 16S rRNA, were quantified via qPCR. All genes quantified here are good candidates for monitoring the dissemination of ARGs from livestock waste. *tet*, *sul*, and *erm* have conferred resistance to three major classes of antibiotics used in animal husbandry (USFDA 2018) via different resistance mechanisms and are thus the most frequently detected ARG classes in livestock waste (He et al. 2020). Additionally, *int*I1 is a mobile genetic element that can be transferred via horizontal gene transfer (HGT) and is a known proxy for anthropogenic pollution (Gillings et al. 2015; Gillings 2018). All samples were analyzed with respect to per gram of dry soil and per 16S rRNA gene copies, and correlation coefficients between individual ARGs and *intI*1 copies were calculated. Results were cross-referenced with package labeling to determine if existing indicators and/or animal manure sources may be correlated to ARG loading.

## Materials and methods

### Sample collection

All commercially available garden products chosen for this survey were purchased in-person from a manufacturer or retailer at major garden and hardware stores. These garden products were sold internationally (*n*=6), nationally (*n*=23), at select states (*n*=1), or locally (*n*=4). Garden products specialized to specific plants (i.e., orchid and rose) were avoided when possible with preference given to garden products branded for general use. Garden products were classified into five major categories: potting soil (*n*=10), garden soil (*n*=7), fruit amendment (*n*=4), lawn amendment (*n*=4), manure (*n*=6), and compost (*n*=3). Garden products spanned 16 brands and included animal manure sources ranging from poultry litter and dairy cow manure to bat guano (manure was included as a listed ingredient in items not labelled as “Manure”). Product informations made available on packaging or online descriptions such as certifications (Demeter or OMRI), labelling, and manure source are summarized in Table 1.

**Table 1:** Certifications, labelling, and manure source for commercially available garden products and soil properties for all samples

Category	Sample	Certifications^a^				Particle size analysis^a^						
		OMRI	Demeter	Labelling^b^	Manure source^c^	% sand	% silt	% clay	% Moisture content^a^	% total solids^a^	% total solids—fixed^a^	% total solids—volatile^a^
Potting soil	POS1	No	No	NA	NA	88.2	10.1	1.7	5.01	94.99	24.19	75.81
	POS2	No	No	O	G	89.3	9.7	1.0	3.84	96.16	23.59	76.41
	POS3	Yes	No	O,P	P	93.0	5.4	1.6	4.16	95.84	45.99	54.01
	POS4	No	No	NA	NA	86.5	13.5	0.0	5.48	94.52	26.80	73.20
	POS5	No	No	O,N	NA	93.2	6.8	0.0	4.66	95.34	27.28	72.72
	POS6	No	No	NA	D	85.9	12.9	1.3	2.86	97.14	45.61	54.39
	POS7	No	No	O,N	P	86.1	13.2	0.7	5.28	94.72	21.35	78.65
	POS8	No	No	NA	NA	95.3	1.9	2.8	3.68	96.32	32.13	67.87
	POS9	No	Yes	O	D	97.3	2.7	0.0	4.75	95.25	54.70	45.30
	POS10	No	No	O,N	NA	91.2	6.1	2.6	4.25	95.75	50.58	49.42
Garden soil	GS1	Yes	No	O,N	P,G	89.1	5.6	5.3	4.03	95.97	43.52	56.48
	GS2	Yes	No	O,N	P	82.6	10.6	6.8	5.80	94.20	26.61	73.39
	GS3	Yes	No	O,N,P	NA	86.2	7.4	6.4	3.60	96.40	21.12	78.88
	GS4	Yes	No	O	NA	96.1	1.1	2.8	5.27	94.73	61.17	38.83
	GS5	No	No	NA	NA	89.0	7.9	3.2	5.06	94.94	26.46	73.54
	GS6	No	No	NA	NA	91.5	4.2	4.3	4.38	95.62	40.14	59.86
	GS7	No	No	NA	NA	85.1	6.4	8.5	4.62	95.38	24.09	75.91
Fruit amendment	FA1	Yes	No	O	P	84.3	8.1	7.7	0.94	99.06	41.68	58.32
	FA2	No	No	O	P	77.4	6.9	15.7	0.77	99.23	43.24	56.76
	FA3	Yes	No	O	P	79.6	3.8	16.6	1.04	98.96	65.17	34.83
	FA4	Yes	No	O,N	P	66.4	14.1	19.4	0.58	99.42	44.42	55.58
Lawn amendment	LA1	Yes	No	O	P	81.2	8.8	10.0	3.99	96.01	42.70	57.30
	LA2	No	No	NA	NA	NA	NA	NA	NA	NA	NA	NA
	LA3	No	No	O	NA	NA	NA	NA	NA	NA	NA	NA
	LA4	Yes	No	O	P	NA	NA	NA	NA	NA	NA	NA
Manure	M1	No	No	N	S	90.0	1.6	8.3	3.26	96.74	66.39	33.61
	M2	No	No	NA	S	NA	NA	NA	4.15	95.85	40.14	59.86
	M3	No	No	O	S	NA	NA	NA	3.72	96.28	36.62	63.38
	M4	Yes	No	O	P	90.5	3.8	5.7	3.76	96.24	59.70	40.30
	M5	No	No	NA	P	NA	NA	NA	NA	NA	NA	NA
	M6	No	No	O	P	NA	NA	NA	3.00	97.00	42.51	57.49
Compost	C1	No	No	O	NA	NA	NA	NA	4.99	95.01	31.92	68.08
	C2	NA	NA	NA	NA	NA	NA	NA	4.94	95.06	68.43	31.57
	C3	No	Yes	NA	D	96.6	1.6	1.9	2.34	97.66	75.52	24.48
Recently landscaped soil	RL1	NA	NA	NA	NA	NA	NA	NA	2.44	97.56	84.58	15.42
	RL2	NA	NA	NA	NA	NA	NA	NA	4.96	95.04	47.09	52.91
	RL3	NA	NA	NA	NA	NA	NA	NA	4.44	95.56	34.29	65.71
Native soil	NS1	NA	NA	NA	NA	NA	NA	NA	1.38	98.62	88.16	11.84
	NS2	NA	NA	NA	NA	NA	NA	NA	1.46	98.54	94.44	5.56
	NS3	NA	NA	NA	NA	NA	NA	NA	1.99	98.01	91.07	8.93
	NS4	NA	NA	NA	NA	NA	NA	NA	1.49	98.51	96.22	3.78
	NS5	NA	NA	NA	NA	NA	NA	NA	1.55	98.45	95.59	4.41

^a^Not available (NA). ^b^ Natural (N), organic (O), premium (P), and not available (NA). ^c^ Dairy cow (D), bat guano (G), poultry (P), steer (S), and not available (NA)

**Fig. 1:**
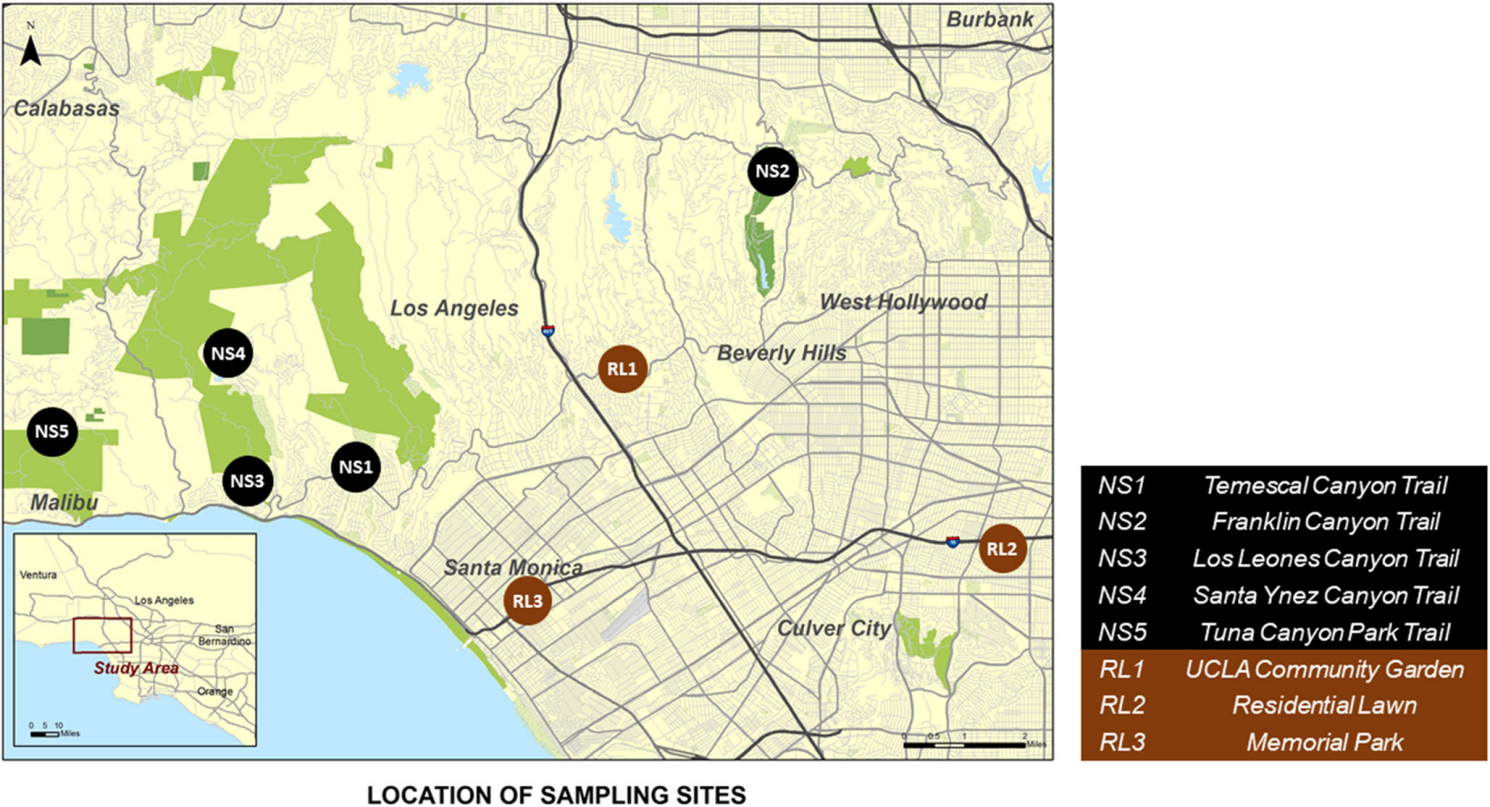
Map of locations where native soils (black) and recently landscaped soils (brown) were collected

The five native soils were collected throughout January 2019 from relatively remote hiking trails selected based on accessibility, foot traffic, and distance to potential anthropogenic contamination sources such as freeways, industrial sites, farms, and residential areas (Fig. 1). For each hiking trail, 1-m^2^ plots were randomly selected approximately 3 ft away from the trail where the soil was undisturbed and uncompacted at the beginning, middle, and end of the trail (*n*=15). Within each 1-m^2^ plot, we collected 10 subsamples from different areas from the top 0–2 in. of soil using sterile 50-mL falcon tubes and spatulas. Soil samples were transported in coolers with ice before being stored at 4°C.

The three recently landscaped soils were collected throughout January 2019 from a community garden in Westwood (RL1), a residential lawn in Crenshaw (RL2), and a park in Santa Monica (RL3) (Fig. 1). For each site, three 1-m^2^ plots were randomly selected (*n*=9). Within each 1-m^2^ plot, we collected 10 subsamples from different areas from the top 0–2 in. of soil using sterile 50-mL falcon tubes and spatulas. The samples were transported in coolers with ice and stored at 4°C.

For processing, soil samples were mixed and triplicate subsamples of 0.25 ± 0.01 g (wet wt.) were measured into sterile 2-mL screwcap tubes loaded with 1.00 ± 0.05 g of 0.7-mm garnet beads (Qiagen, Germantown, MD) for gene quantification analysis. Screwcap tubes were stored at −80°C until DNA extraction. Additionally, triplicate subsamples of 90.00 ± 2.00 g (wet wt.) were measured into sterile glass sample jars and stored at −20 °C for soil characterization.

### DNA extraction and qPCR

All DNA extractions were completed within 2 weeks of sample collection using the DNeasy PowerSoil Kit (Qiagen, Germantown, MD) per the manufacturer’s guidelines except for the cell lysis step where a BioSpec Mini-BeadBeater-8 (BioSpec Products, Bartlesville, OK) was used for 2 min, in place of vortexing for 10 min. All samples were analyzed for *sul*1, *sul*2, *tet*(L), *tet*(W), *erm*(F), *intI*1, and 16S rRNA via qPCR with a StepOne Plus (Applied Biosystems, Foster City, CA). Primers and primer concentrations (Table S1) and reaction specifics (Table S2) were validated previously in the literature (Ji et al. 2012; Knapp et al. 2010; Luo et al. 2010; Pei et al. 2006; Zhou et al. 2014). Standard curves were designed using sequences obtained through the National Center for Biotechnology (NCBI) database and ordered through IDT Technologies (Echeverria-Palencia et al. 2017).

### Soil characterization

Sand, silt, and clay distributions were obtained using the particle size analysis hydrometer method. Triplicate subsamples of 80.00 ± 1.00 g (wet wt.) were oven dried at 70°C for 24 h. For each triplicate subsample, 50.00 ± 1.00 g (dry wt.) were measured into a beaker. One hundred milliliters of a 5% (w/w) sodium metaphosphate solution and 200-mL of deionized (DI) water were added to the beaker to mix at 125 rpm for 24 h. The contents in the beaker were transferred to 1-L cylinders and DI water was added up to the 1 L mark. The contents in the cylinders were mixed, and temperature and hydrometer readings were taken at 40 s to obtain % sand. Without disturbing the cylinders, temperature and hydrometer readings were taken again at 2 h to obtain % clay. The % silt was obtained by subtracting % sand and % clay from 100% (Ashworth et al. 2001).

To obtain moisture content and total solids, triplicate subsamples of 2.00 ± 0.05 g were oven dried at 105°C for 24 h. To determine the fractions of total solids-fixed and total solids-volatile, the dried subsamples were ignited in a furnace at 550°C for 2 h. (EPA 2001). NA in Table 1 indicates insufficient amount of sample for triplicate subsamples to be analyzed.

### Statistics

Microsoft Excel was used to calculate triplicate subsample absolute and relative gene abundances from raw thermocycler data. Average absolute and relative gene abundances were calculated from triplicate subsamples for each sample, category, and gene. The SAS CORR procedure was used to calculate the Pearson correlation coefficients between gene abundances and between gene abundances and soil characteristics. R Studio was used to determine normality for each category and each gene through visual observation of histograms and quantile-quantile (Q-Q) plots. The dataset was found to contain non-normal distributions. The Wilcoxon signed-rank test was used to compare garden products and native soils, OMRI and non-OMRI certified garden products, and manure sourcing and non-manure sourcing.

## Results and discussion

### Gene quantities

**Fig. 2:**
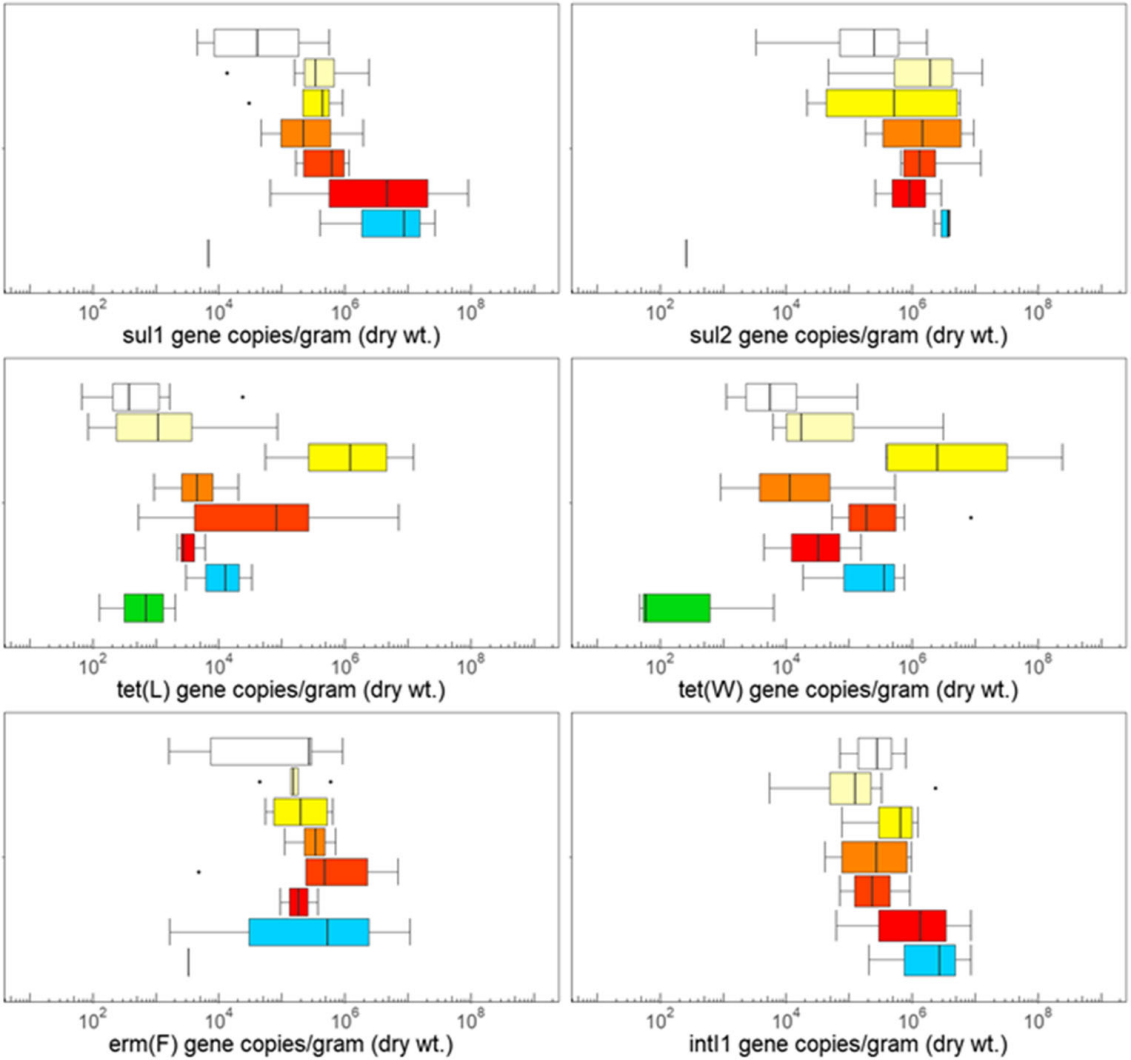
Absolute gene abundances for potting soil (white, *n*=10), garden soil (light yellow, *n*=7), fruit amendment (yellow, *n*=4), lawn amendment (light orange, *n*=4), manure (orange, *n*=6), compost (red, *n*=3), recently landscaped soil (blue, *n*=3), and native soil (green, *n*=5)

**Fig. 3:**
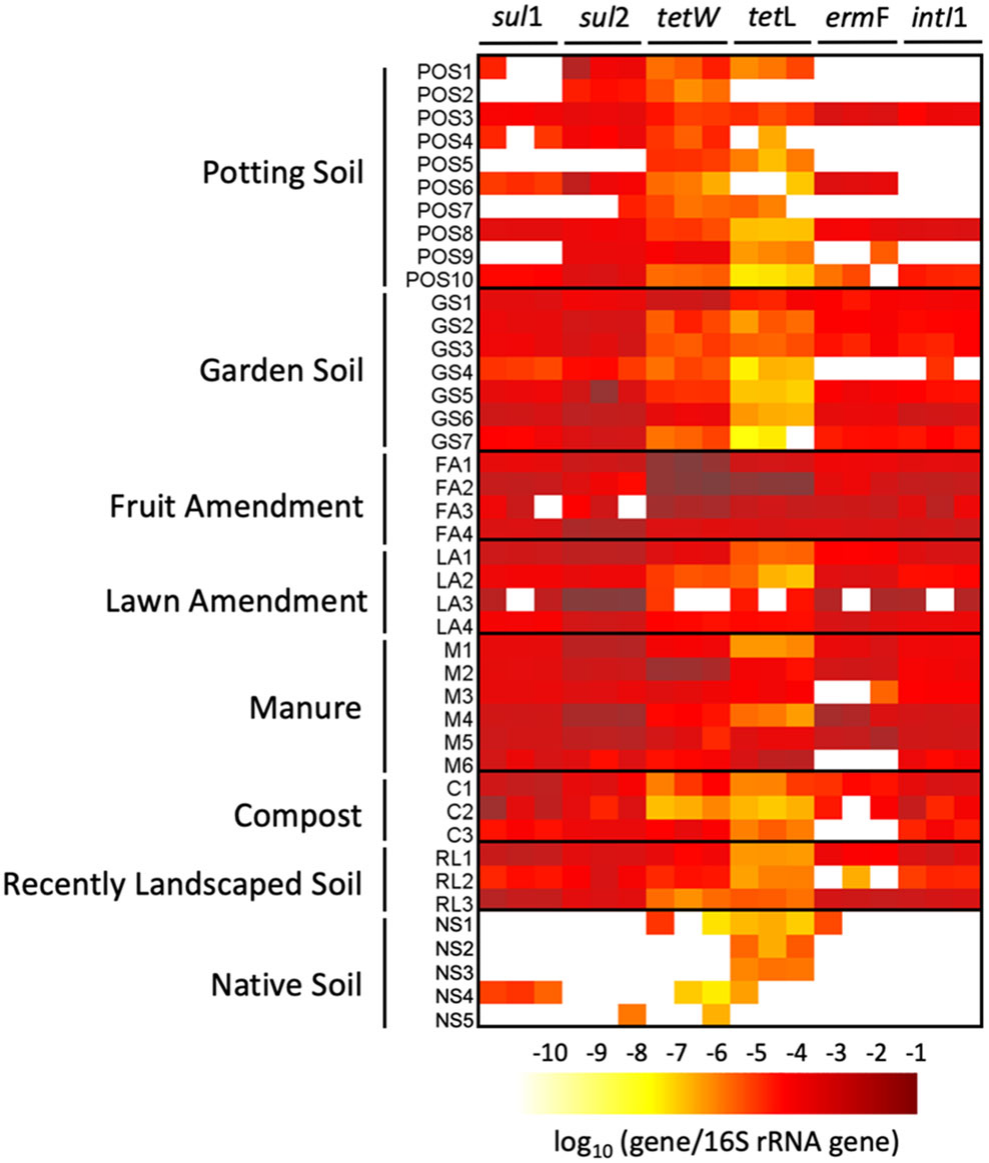
Heatmap of relative gene abundances for 10 potting soils, 7 garden soils, 4 fruit amendments, 4 lawn amendments, 6 manures, 3 composts, 3 recently landscaped soils, and 5 native soils

*sul*, *tet*, and *erm* confer resistance to sulfonamides, tetracyclines, and macrolides, respectively, three major classes of antibiotics approved for use in livestock (USFDA 2018). *intI*1 is a useful indicator of anthropogenic pollution (Gillings et al. 2015; Gillings 2018) and 16S rRNA is a total bacteria surrogate measure. Quantities of ARGs and *intI*1 for commercially available garden products, recently landscaped soils, and native soils can be found in Figs. 2 and 3 and Table S3-6.

*sul*1 was detected in 30 out of 34 garden products, 3 out of 3 recently landscaped soils, and 1 out of 5 native soils. Mean absolute and relative gene quantities were roughly three orders of magnitude higher in garden products (10^6^ gene copies/gram and 10^−2^ gene copies/16S rRNA gene copies) than in native soils (10^3^ gene copies/gram and 10^−5^ gene copies/16S rRNA gene copies). *sul*2 was detected in 33 out of 34 garden products, 3 out of 3 recently landscaped soils, and 1 out of 5 native soils. *sul*2 mean gene abundances showed a four order magnitude difference between garden products (10^6^ gene copies/gram and 10^−2^ gene copies/16S rRNA gene copies) and native soils (10^2^ gene copies/gram and 10^−6^ gene copies/16S rRNA gene copies).

*tet*(L) was detected in 34 out of 34 garden products, 3 out of 3 recently landscaped soils, and 4 out of 5 native soils. Mean absolute and relative abundances of the gene were three orders of magnitude higher in garden products (10^6^ gene copies/gram and 10^−2^ gene copies/16S rRNA gene copies) in comparison to native soils (10^3^ gene copies/gram and 10^−5^ gene copies/16S rRNA gene copies). *tet*(W) was detected in all garden products, 3 out of 3 recently landscaped soils, and 3 out of 5 native soils. *tet*(W) mean gene quantities were high in garden products (10^7^ gene copies/gram and 10^−1^ gene copies/16S rRNA gene copies) and low in native soils (10^3^ gene copies/gram and 10^−5^ gene copies/16S rRNA gene copies).

*erm*(F) quantities were detected in 26 out of 34 garden products, 3 out of 3 recently landscaped soils, and 1 out of the 5 native soils. While *erm*(F) mean abundances in garden products were 10^6^ gene copies/gram and 10^-2^ gene copies/16S rRNA gene copies, in native soils they were 10^3^ gene copies/gram and 10^-5^ gene copies/16S rRNA gene copies.

*intI*1 was detected in 29 out of 34 garden products and in 3 out of 3 recently landscaped soils. While the mean absolute and relative concentrations in garden products were 10^6^ gene copies/gram and 10^−3^ gene copies/16S rRNA gene copies, respectively, *intI*1 was undetected in native soils.

Relative gene abundances in commercial garden products are comparable to soils amended with manure-based commercial organic fertilizers (COFs) (Zhou et al. 2017). This may be attributed to COFs introducing approximately 60–70% of ARGs to soils amended with COFs (Zhou et al. 2019). Findings here are also in accord with relative gene abundances in poultry manure (Cheng et al. 2013), and in soils amended with dairy manure (Dungan et al. 2019; McKinney et al. 2018; Munir and Xagoraraki 2011) and composted dairy manure (Tien et al. 2017; Jacobs et al. 2019). Additionally, relative gene quantities in soils from 6 Los Angeles parks (Echeverria-Palencia et al. 2017) are more comparable to the recently landscaped soils than the native soils.

### Inter-gene observations

In the present study, the absolute quantities of *intI*1 and *sul*1 were found to exhibit a strong positive correlation (*r* = 0.9648, *p* < 0.0001). When analyzing relative abundances, *intI*1 was found to be highly correlated with *sul*2 (*r* = 0.8505, *p* < 0.0001) and slightly correlated with *erm*(F) (*r* = 0.52623, *p* < 0.0001). Additionally, *erm*(F) and *sul*2 were also slightly correlated (*r* = 0.5804, *p* < 0.0001) (Table S7). Several environmental studies have found strong associations between *intI*1 and *sul*1 gene quantities (Gillings et al. 2015; Lin et al. 2016; Nardelli et al. 2012; Peng et al. 2017), attributable to *intI*1 and *sul*1 being components of class 1 integrons (Gillings et al. 2008; Gillings et al. 2015). Correlations of *intI*1 with *sul*2 (Lin et al. 2016; Peng et al. 2017) and *erm*(F) (Peng et al. 2017) have also been reported for fertilized soils.

### Garden products versus native soils

A Wilcoxon test for difference between the means of the native soils and garden products were significant (*p* < 0.05) for all absolute and relative gene quantities except *tet*(L). The *p*-values for absolute gene quantities are 0.003, 0.001, 0.011, 0.078, 0.002, and 0.004 for *sul*1, *sul*2, *erm*(F), *tet*(L), *tet*(W), and *intI*1, respectively. The *p*-values for relative gene quantities are 0.003, 0.001, 0.013, 0.115, 0.001, and 0.004 for *sul*1, *sul*2, *erm*(F), *tet*(L), *tet*(W), and *intI*1, respectively (Table S7). These results indicate that the garden products are a source of ARGs when compared to native soils.

### Certifications and genes

With 33% of garden products listed as OMRI approved, OMRI was the most advertised certification. OMRI-certified and not certified garden products were found to contain gene quantities that were generally comparable (*p* > 0.05) (Table S7), indicating that the OMRI certification cannot serve as an indicator for ARG introduction via garden products.

Demeter certification applies exclusively to biodynamic farms and corresponded to two garden products from just one brand (POS9 and C3). More garden products of this certification are needed to determine if the Demeter certification may serve as an ARG predictor.

### Manure sourcing and genes

A Wilcoxon test for difference between the means of manured and non-manured sources proved to be insignificant (*p* > 0.05) except for *tet*(L) and *tet*(W) (Table S7). However, manure source information was not consistently available across gardening products. Even when manure source was available, proportions and pretreatments of manure were considered proprietary, largely limiting the ability to screen for pre-treatment effects on final garden product ARG levels.

Results in this this study indicate that regulations considering product labelling, pre-treatment, and antibiotic, ARG, and ARB loading are needed.

## Supplementary Information

ESM 1 (DOCX 49 kb)

## Data Availability

All data generated or analyzed during this study are included in this published article and its supplementary information file.

## References

[CR1] Allen HK, Donato J, Wang HH, Cloud-Hansen KA, Davies J, Handelsman J (2010). Call of the wild: Antibiotic resistance genes in natural environments. Nat Rev Microbiol.

[CR2] Ashworth J, Keyes D, Kirk R, Lessard R (2001). Standard procedure in the hydrometer method for particle size analysis. Commun Soil Sci Plant Anal.

[CR3] Cheng W, Chen H, Su C, Yan S (2013). Abundance and persistence of antibiotic resistance genes in livestock farms: A comprehensive investigation in eastern China. Environ Int.

[CR4] Code of Federal Regulations https://www.ecfr.gov/cgi-bin/text-idx?SID=4835b8a48e2533555547c742ae912ed2&mc=true&node=sp7.3.205.d&rgn=div6#se7.3.205_1300. Accessed August 2020

[CR5] Dungan RS, Strausbaugh C, Leytem A (2019). Survey of selected antibiotic resistance genes in agricultural and non-agricultural soils in South-Central Idaho. FEMS Microbiol Ecol.

[CR6] Echeverria-Palencia CM, Thulsiraj V, Tran N, Ericksen CA, Melendez I, Sanchez MG, Walpert D, Yuan T, Ficara E, Senthilkumar N, Sun F, Li R, Hernandez-Cira M, Gamboa D, Haro H, Paulson SE, Zhu Y, Jay JA (2017). Disparate antibiotic resistance gene quantities revealed across 4 major cities in California: a survey in drinking water, air, and soil at 24 public parks. ACS Omega.

[CR7] EPA. (2001) METHOD 1684 Total, Fixed, and Volatile Solids in Water , Solids , and Biosolids Draft January 2001 U. S . Environmental Protection Agency Office of Water Office of Science and Technology Engineering and Analysis Division ( 4303 ), (January), 1–13.

[CR8] Fahrenfeld N, Knowlton K, Krometis LA, Hession WC, Xia K, Lipscomb E, Libuit K, Green BL, Pruden A (2014). Effect of manure application on abundance of antibiotic resistance genes and their attenuation rates in soil: field-scale mass balance approach. Environ Sci Technol.

[CR9] Gillings MR (2018). DNA as a pollutant: the clinical class 1 integron. Curr Pollut Rep.

[CR10] Gillings M, Boucher Y, Labbate M, Holmes A, Krishnan S, Holley M, Stokes HW (2008). The evolution of class 1 integrons and the rise of antibiotic resistance. J Bacteriol.

[CR11] Gillings MR, Gaze WH, Pruden A, Smalla K, Tiedje JM, Zhu Y-G (2015). Using the class 1 integron-integrase gene as a proxy for anthropogenic pollution. ISME J.

[CR12] Hall BG, Barlow M (2004). Evolution of the serine β-lactamases: past, present and future. Drug Resist Updat.

[CR13] He Y, Yuan Q, Mathieu J, Stadler L, Senehi N, Sun R, Alvarez PJJ (2020). Antibiotic resistance genes from livestock waste: occurrence, dissemination, and treatment. npj Clean Water.

[CR14] Heuer H, Schmitt H, Smalla K (2011). Antibiotic resistance gene spread due to manure application on agricultural fields. Curr Opin Microbiol.

[CR15] Heuer H, Solehati Q, Zimmerling U, Kleineidam K, Schloter M, Müller T, Focks A, Thiele-Bruhn S, Smalla K (2011). Accumulation of sulfonamide resistance genes in arable soils due to repeated application of manure containing sulfadiazine. Appl Environ Microbiol.

[CR16] Jacobs K, Wind L, Krometis L-A, Hession WC, Pruden A (2019). Fecal indicator bacteria and antibiotic resistance genes in storm runoff from dairy manure and compost-amended vegetable plots. J Environ Qual.

[CR17] Jechalke S, Kopmann C, Rosendahl I, Groeneweg J, Weichelt V, Krögerrecklenfort E, Brandes N, Nordwig M, Ding G-C, Siemens J, Heuer H, Smalla K (2013). Increased abundance and transferability of resistance genes after field application of manure from sulfadiazine-treated pigs. Appl Environ Microbiol.

[CR18] Ji X, Shen Q, Liu F, Ma J, Xu G, Wang Y, Wu M (2012). Antibiotic resistance gene abundances associated with antibiotics and heavy metals in animal manures and agricultural soils adjacent to feedlots in Shanghai, China. J Hazard Mater.

[CR19] Knapp CW, Dolfing J, Ehlert PAI, Graham DW (2010). Evidence of increasing antibiotic resistance gene abundances in archived soils since 1940. Environ Sci Technol.

[CR20] Lin H, Sun W, Zhang Z, Chapman SJ, Freitag TE, Fu J, Zhang X, Ma J (2016). Effects of manure and mineral fertilization strategies on soil antibiotic resistance gene levels and microbial community in a paddy-upland rotation system. Environ Pollut.

[CR21] Liu J, Zhao Z, Orfe L, Subbiah M, Call DR (2016). Soil-borne reservoirs of antibiotic-resistant bacteria are established following therapeutic treatment of dairy calves. Environ Microbiol.

[CR22] Looft T, Johnson TA, Allen HK, Bayles DO, Alt DP, Stedtfeld RD, Sul WJ, Stedtfeld TM, Chai B, Cole JR, Hashsham SA, Tiedje JM, Stanton TB (2012). In-feed antibiotic effects on the swine intestinal microbiome. Proc Natl Acad Sci.

[CR23] Luo YI, Mao D, Rysz M (2010). Trends in Antibiotic Resistance Genes Occurrence in the Haihe River, China. Environ Sci Technol.

[CR24] Marti R, Scott A, Tien YC, Murray R, Sabourin L, Zhang Y, Topp E (2013). Impact of manure fertilization on the abundance of antibiotic-resistant bacteria and frequency of detection of antibiotic resistance genes in soil and on vegetables at harvest. Appl Environ Microbiol.

[CR25] McEachran A (2012). Antibiotics, bacteria, and antibiotic resistance genes: aerial transport from cattle feed yards via particulate matter. Environ Health Perspect.

[CR26] McKinney C, Dungan R, Moore A, Leytem A (2018). Occurrence and abundance of antibiotic resistance genes in agricultural soils receiving dairy manure. FEMS Microbiol Ecol.

[CR27] Munir M, Xagoraraki I (2011). Levels of antibiotic resistance genes in manure, biosolids, and fertilized soil. J Environ Qual.

[CR28] Nardelli M, Scalzo PM, Ramírez MS, Quiroga MP, Cassini MH, Centrón D (2012). Class 1 Integrons in environments with different degrees of urbanization. PLoS One.

[CR29] National Organic Program Handbook https://www.ams.usda.gov/sites/default/files/media/Program%20Handbk_TOC.pdf. Accessed August 2020

[CR30] O’Neill J (2014) Antimicrobial Resistance: tackling a crisis for the health and wealth of nations. Rev Antimicrob Resist Chaired. (December).

[CR31] Pei R, Kim S-C, Carlson KH, Pruden A (2006). Effect of river landscape on the sediment concentrations of antibiotics and corresponding antibiotic resistance genes (ARG). Water Res.

[CR32] Peng S, Feng Y, Wang Y, Guo X, Chu H, Lin X (2017). Prevalence of antibiotic resistance genes in soils after continually applied with different manure for 30 years. J Hazard Mater.

[CR33] Sancheza HM, Echeverria C, Thulsiraj V, Zimmer-Faust A, Flores A, Laitz M, Healy G, Mahendra S, Paulson SE, Zhu Y (2016). Antibiotic resistance in airborne bacteria near conventional and organic beef cattle farms in California, USA. Water Air Soil Pollut.

[CR34] Sandberg KD, LaPara TM (2016). The fate of antibiotic resistance genes and class 1 integrons following the application of swine and dairy manure to soils. FEMS Microbiol Ecol.

[CR35] Sarmah AK, Meyer MT, Boxall ABA (2006). A global perspective on the use, sales, exposure pathways, occurrence, fate and effects of veterinary antibiotics (VAs) in the environment. Chemosphere..

[CR36] Tien YC, Li B, Zhang T, Scott A, Murray R, Sabourin L, Marti R, Topp E (2017). Impact of dairy manure pre-application treatment on manure composition, soil dynamics of antibiotic resistance genes, and abundance of antibiotic-resistance genes on vegetables at harvest. Sci Total Environ.

[CR37] USFDA. (2018) Antimicrobials Sold or Distributed for Use in Food-Producing Animals

[CR38] Wang L, Gutek A, Grewal S, Michel FC, Yu Z (2015). Changes in diversity of cultured bacteria resistant to erythromycin and tetracycline in swine manure during simulated composting and lagoon storage. Lett Appl Microbiol.

[CR39] Zhou T, Lu J, Tong Y, Li S, Wang X (2014). Distribution of antibiotic resistance genes in Bosten Lake, Xinjiang, China. Water Sci Technol.

[CR40] Zhou X, Qiao M, Wang FH, Zhu YG (2017). Use of commercial organic fertilizer increases the abundance of antibiotic resistance genes and antibiotics in soil. Environ Sci Pollut Res.

[CR41] Zhou X, Qiao M, Su JQ, Zhu YG (2019). High-throughput characterization of antibiotic resistome in soil amended with commercial organic fertilizers. J Soils Sediments.

