# Supplementary material for: Commercially available garden products as important sources of antibiotic resistance genes—a survey
Source: Environ Sci Pollut Res Int. 2021 Apr 9;28(32):43507–14. doi: 10.1007/s11356-021-13333-7 (PMC8357637; doi:10.1007/s11356-021-13333-7)
Supplement: Supplementary file 1 — (DOCX 49 kb) [file 11356_2021_13333_MOESM1_ESM.docx]

***Environmental Science and Pollution Research***

**Supplemental Information**

**Commercially available garden products as important sources of antibiotic resistance genes—A survey**

Marisol Cira^1,3^, Cristina M. Echeverria-Palencia^1,3^, Ileana Callejas^1^, Karina Jimenez^1^, Rafael Herrera Jr.^1^, Wei-Cheng Hung^1^, Nicolas Colima^1^, Amanda Schmidt^1^, and Jennifer A. Jay^1,2,4^

^1^ University of California Los Angeles, Civil and Environmental Engineering Department, 420 Westwood Plaza, Los Angeles, CA 90095

^2^ University of California Los Angeles, Institute of the Environment and Sustainability, LaKretz Building, Los Angeles, CA 90095

^3^ Both authors contributed equally to this work.

^4^ jjay@seas.ucla.edu, 310-866-2444

| DNA Extraction and qPCR | 1 |
| --- | --- |
| Table S1. Forward and reverse primers for qPCR assays | 2 |
| Table S2. qPCR reaction conditions | 3 |
| Table S3. Average Log_10_ gene abundances (gene copies/gram (dry wt.)) for product categories | 3 |
| Table S4. Average Log_10_ gene abundances (gene copies/16S rRNA gene copies) for product categories | 3 |
| Table S5. Average Log_10_ gene abundances (gene copies/gram (dry wt.)) for samples  Table S6. Average Log_10_ gene abundances (gene copies/16S rRNA gene copies) for samples | 4  5 |

Table S7. P-values from Wilcoxon hypothesis tests 6

**DNA Extraction and qPCR**

Every extraction included an additional 2 mL screw cap tube preloaded with 1.00 ± 0.05 g of 0.7 mm diameter garnet beads as an extraction blank to confirm absence of contamination during the extraction process. Eluted DNA was aliquoted and stored at -20 °C awaiting qPCR analysis. Total DNA concentration was measured using UV absorption via a Nanodrop 2000C (Thermo Scientific, Waltham MA), as were 260/280 absorbance ratios.

All assays consisted of 40 cycles and were conducted using PowerUp SYBR Green Master Mix and consisted of: 12.5 μL of PowerUp SYBR Green MasterMix (Life Technologies, Grand Island, NY), 1.25 μL of each primer, 2 μL of template DNA, and 8 μL of molecular grade water. Primers and primer concentrations and reaction specifics as validated previously in the literature can be found in Table S1 and Table S2 (Ji et al. 2012; Knapp et al. 2010; Luo et al. 2010; Pei et al. 2006; Zhou et al. 2014). Each assay run included a 7-point standard curve positive control, applicable extraction blanks, and a negative control of molecular grade water, with each sample plated in triplicate wells.

Well spike and serial dilution tests were used to determine the appropriate DNA concentration and dilutions were accomplished using molecular grade water. Serial dilution involved picking random samples and serially diluting DNA by a factor of 4 and performing qPCR on all dilutions to determine the ideal dilution factor. All study samples were quantified for DNA concentration and the dilution factor was used in conjunction with the lowest DNA yield to determine an appropriate standardized dilution concentration. A second set of randomly selected samples were then used to verify absence of inhibition through performing well spikes. Unquantifiable samples were run at higher concentrations to verify absence of gene and ensure that over-dilution was not occurring.

Standard curves were designed using sequences obtained through the NCBI database and ordered through IDT Technologies (Echeverria-Palencia et al. 2017). Known concentrations of the designed DNA fragment were run alongside environmental samples, yielding a seven-point standard curve and allowing for quantitation of gene copies. Standard curve efficiencies were at or above 91% across all qPCR assays and all R^2^ values were at or above 0.99. Melt curves were used to further verify target gene amplification specificity.

**Table S1**. Forward and reverse primers for qPCR assays.

| Target Gene | Primer | Concentration (nM) | Sequence (5'-3') | Amplicon Size (bp) |
| --- | --- | --- | --- | --- |
| *sul*1^a^ | *sul*1-F | 200 | CGCACCGGAAACATCGCTGCAC | 258 |
|  | *sul*1-R |  | TGAAGTTCCGCCGCAAGGCTCG |  |
| *sul*2^b^ | *sul*2-F | 200 | CTCCGATGGAGGCCGGTAT | 449 |
|  | *sul*2-R |  | GGGAATGCCATCTGCCTTGA |  |
| *tet*(L)^c^ | *tet*(L)-F | 900 | GGTTTTGAACGTCTCATTACCTGAT | 250 |
|  | *tet*(L)-R |  | CCAATGGAAAAGGTTAACATAAAGG |  |
| *tet*(W)^d^ | *tet*(W)-F | 200 | GAG AGC CTG CTA TAT GCC AGC | 385 |
|  | *tet*(W)-R |  | GGG CGT ATC CAC AAT GTT AAC |  |
| *erm*(F)^c^ | *erm*(F)-F | 500 | TCGTTTTACGGGTCAGCACTT | 246 |
|  | *erm*(F)-R |  | CAACCAAAGCTGTGTCGTTT |  |
| *int*I1^b^ | *int*I1-F | 200 | GGCTTCGTGATGCCTGCTT | 424 |
|  | *int*I1-R |  | CATTCCTGGCCGTGGTTCT |  |
| 16S rRNA^e^ | 16S rRNA-F | 100 | CCTACGGGAGGCAGCAG | 257 |
|  | 16S rRNA-R |  | ATTACCGCGGCTGCTGG |  |

^a^Pei et al. 2006 ^b^Luo et al. 2010 ^c^Knapp et al. 2010 ^d^Zhou et al. 2014 ^e^Ji et al. 2012

**Table S2.** qPCR reaction conditions.

| Target Gene | Holding | | Denaturation | | Annealing | | Extension | | R^2^ | Amp. Eff. |
| --- | --- | --- | --- | --- | --- | --- | --- | --- | --- | --- |
|  | Temp. (℃) | Time (min) | Temp. (℃) | Time (s) | Temp. (℃) | Time (s) | Temp. (℃) | Time (s) |  |  |
| *sul*1^a^ | 95 | 10 | 95 | 15 | 65 | 30 | 72 | 30 | 0.991 ± 0.020 | 97 ± 5.8 |
| *sul*2^b^ | 95 | 15 | 95 | 15 | 58.5 | 30 | 72 | 30 | 0.992 ± 0.010 | 91 ± 0.8 |
| *tet*(L)^c^ | 95 | 10 | 95 | 15 | 60 | 30 | - | - | 1.000 ± 0.000 | 97 ± 2.0 |
| *tet*(W)^d^ | 95 | 15 | 95 | 15 | 60 | 30 | 72 | 30 | 0.995 ± 0.001 | 98 ± 3.5 |
| *erm*(F)^c^ | 95 | 10 | 94 | 20 | 60 | 60 | - | - | 0.999 ± 0.001 | 97 ± 1.9 |
| *int*I1^b^ | 95 | 10 | 95 | 15 | 55 | 30 | 72 | 30 | 0.999 ± 0.000 | 98 ± 2.2 |
| 16S rRNA^e^ | 95 | 15 | 95 | 15 | 60 | 30 | 72 | 30 | 0.999 ± 0.005 | 97 ± 2.1 |

^a^Pei et al. 2006 ^b^Luo et al. 2010 ^c^Knapp et al. 2010 ^d^Zhou et al. 2014 ^e^Ji et al. 2012

**Table S3.** Average Log_10_ gene abundances (gene copies/gram (dry wt.)) for product categories.

| Category |  | *sul*1 | | *sul*2 | | *tet*(L) | | *tet*(W) | | *erm*(F) | | *intI*1 | |
| --- | --- | --- | --- | --- | --- | --- | --- | --- | --- | --- | --- | --- | --- |
|  | n | Mean | Max | Mean | Max | Mean | Max | Mean | Max | Mean | Max | Mean | Max |
| Potting Soil | 10 | 5.0 | 5.7 | 5.7 | 6.2 | 3.4 | 4.4 | 4.3 | 5.1 | 5.2 | 6.0 | 5.1 | 5.9 |
| Garden Soil | 7 | 5.8 | 6.4 | 6.6 | 7.1 | 4.1 | 7.1 | 5.7 | 6.5 | 5.3 | 5.8 | 5.6 | 6.4 |
| Fruit Amend. | 4 | 5.7 | 6.0 | 6.4 | 6.8 | 6.6 | 7.1 | 8.1 | 8.4 | 5.5 | 5.8 | 5.8 | 6.1 |
| Lawn Amend. | 4 | 5.8 | 6.3 | 6.6 | 7.0 | 3.9 | 4.3 | 5.2 | 5.7 | 5.6 | 5.9 | 5.7 | 6.0 |
| Manure | 6 | 5.8 | 6.1 | 6.5 | 7.1 | 6.1 | 6.9 | 6.2 | 6.9 | 6.2 | 6.8 | 5.5 | 6.0 |
| Compost | 3 | 7.5 | 8.0 | 6.1 | 6.5 | 3.6 | 3.8 | 4.8 | 5.2 | 5.2 | 5.6 | 6.5 | 6.9 |
| Recently Landscaped Soil | 3 | 7.1 | 7.4 | 6.5 | 6.6 | 4.2 | 4.5 | 5.6 | 5.9 | 6.6 | 7.0 | 6.6 | 6.9 |
| Native Soil | 5 | 3.1 | 3.8 | 1.7 | 2.4 | 2.9 | 3.3 | 3.1 | 3.8 | 2.8 | 3.5 | ND | ND |

**Table S4.** Average Log_10_ gene abundances (gene copies/16S rRNA gene copies) for product categories.

| Category |  | *sul*1 | | *sul*2 | | *tet*(L) | | *tet*(W) | | *erm*(F) | | *intI*1 | |
| --- | --- | --- | --- | --- | --- | --- | --- | --- | --- | --- | --- | --- | --- |
|  | n | Mean | Max | Mean | Max | Mean | Max | Mean | Max | Mean | Max | Mean | Max |
| Potting Soil | 10 | -3.4 | -2.6 | -2.6 | -1.9 | -4.8 | -4.1 | -3.8 | -3.0 | -3.2 | -2.5 | -3.4 | -2.5 |
| Garden Soil | 7 | -2.7 | -2.2 | -1.8 | -1.1 | -4.2 | -1.1 | -2.8 | -2.0 | -3.3 | -2.8 | -2.9 | -2.2 |
| Fruit Amend. | 4 | -2.3 | -1.9 | -1.8 | -1.3 | -1.0 | -0.4 | -0.6 | -0.2 | -2.3 | -2.0 | -2.1 | -1.8 |
| Lawn Amend. | 4 | -2.2 | -1.8 | -0.8 | -0.2 | -3.9 | -3.5 | -3.1 | -2.6 | -2.0 | -1.5 | -2.0 | -1.5 |
| Manure | 6 | -2.4 | -2.1 | -1.6 | -1.1 | -2.5 | -1.8 | -1.7 | -0.9 | -1.9 | -1.4 | -2.6 | -2.2 |
| Compost | 3 | -1.7 | -1.3 | -2.8 | -2.7 | -4.8 | -4.5 | -3.4 | -3.0 | -3.8 | -3.6 | -2.6 | -2.4 |
| Recently Landscaped Soil | 3 | -2.0 | -1.7 | -2.6 | -2.5 | -4.9 | -4.6 | -3.4 | -3.0 | -2.5 | -2.1 | -2.4 | -2.2 |
| Native Soil | 5 | -4.9 | -4.2 | -6.1 | -5.4 | -5.2 | -4.8 | -5.2 | -4.5 | -5.5 | -4.8 | ND | ND |

**Table S5.** Average Log_10_ gene abundances (gene copies/gram (dry wt.)) for samples.

| Category | Sample | *sul*1 | *sul*2 | *tet*(L) | *tet*(W) | *erm*(F) | *intI*1 |
| --- | --- | --- | --- | --- | --- | --- | --- |
| Potting Soil | POS1 | 3.9 | 6.2 | 3.2 | 3.7 | ND | ND |
|  | POS2 | ND | 4.4 | ND | 3.3 | ND | ND |
|  | POS3 | 5.3 | 5.7 | 4.4 | 4.5 | 6.0 | 5.4 |
|  | POS4 | 3.7 | 4.8 | 2.2 | 3.8 | ND | ND |
|  | POS5 | ND | ND | 2.6 | 3.7 | ND | ND |
|  | POS6 | 4.1 | 5.8 | 1.8 | 3.1 | 5.5 | ND |
|  | POS7 | ND | 3.5 | 2.8 | 3.2 | ND | ND |
|  | POS8 | 5.7 | 5.4 | 2.5 | 4.3 | 5.4 | 5.9 |
|  | POS9 | ND | 5.3 | 3.0 | 5.1 | 3.2 | ND |
|  | POS10 | 5.2 | 6.1 | 2.3 | 3.9 | 3.9 | 4.8 |
| Garden Soil | GS1 | 5.9 | 5.6 | 4.9 | 6.5 | 5.1 | 5.5 |
|  | GS2 | 5.7 | 6.3 | 3.5 | 4.2 | 5.3 | 5.1 |
|  | GS3 | 5.2 | 5.8 | 3.6 | 3.8 | 4.6 | 4.5 |
|  | GS4 | 4.1 | 4.7 | 2.4 | 3.8 | ND | 3.7 |
|  | GS5 | 5.5 | 7.1 | 2.4 | 4.4 | 5.2 | 4.9 |
|  | GS6 | 6.4 | 6.8 | 3.0 | 5.7 | 5.8 | 6.4 |
|  | GS7 | 5.5 | 6.5 | 1.9 | 4.2 | 5.1 | 5.2 |
| Fruit Amendment | FA1 | 6.0 | 6.7 | 6.5 | 8.4 | 5.8 | 6.1 |
|  | FA2 | 5.6 | 4.7 | 7.1 | 7.2 | 4.9 | 5.7 |
|  | FA3 | 4.5 | 4.3 | 4.7 | 5.6 | 4.7 | 4.9 |
|  | FA4 | 5.7 | 6.8 | 5.7 | 5.6 | 5.7 | 6.0 |
| Lawn Amendment | LA1 | 6.3 | 6.7 | 3.8 | 5.7 | 5.0 | 6.0 |
|  | LA2 | 5.1 | 5.3 | 3.0 | 3.8 | 5.6 | 4.6 |
|  | LA3 | 5.6 | 7.0 | 3.5 | 3.0 | 5.9 | 5.9 |
|  | LA4 | 4.7 | 5.6 | 4.3 | 4.3 | 5.5 | 5.0 |
| Manure | M1 | 5.2 | 6.4 | 2.7 | 4.9 | 5.4 | 5.0 |
|  | M2 | 5.2 | 5.8 | 4.7 | 6.9 | 5.7 | 4.9 |
|  | M3 | 5.9 | 6.0 | 5.5 | 5.9 | 3.7 | 5.4 |
|  | M4 | 6.0 | 7.1 | 3.2 | 4.7 | 6.8 | 6.0 |
|  | M5 | 5.7 | 6.3 | 5.2 | 5.3 | 6.4 | 5.7 |
|  | M6 | 6.1 | 5.8 | 6.9 | 5.3 | ND | 5.3 |
| Compost | C1 | 6.7 | 6.0 | 3.8 | 4.5 | 5.0 | 6.1 |
|  | C2 | 8.0 | 6.5 | 3.3 | 3.6 | 5.6 | 6.9 |
|  | C3 | 4.8 | 5.4 | 3.4 | 5.2 | ND | 4.8 |
| Recently Landscaped Soil | RL1 | 6.9 | 6.3 | 3.5 | 5.9 | 5.7 | 6.4 |
|  | RL2 | 5.6 | 6.6 | 4.1 | 5.6 | 3.2 | 5.3 |
|  | RL3 | 7.4 | 6.6 | 4.5 | 4.3 | 7.0 | 6.9 |
| Native Soil | NS1 | ND | ND | 2.6 | 3.8 | 3.5 | ND |
|  | NS2 | ND | ND | 3.1 | ND | ND | ND |
|  | NS3 | ND | ND | 3.3 | ND | ND | ND |
|  | NS4 | 3.8 | ND | 2.1 | 1.8 | ND | ND |
|  | NS5 | ND | 2.4 | ND | 1.7 | ND | ND |

**Table S6.** Average Log_10_ gene abundances (gene copies/16S rRNA gene copies) for samples.

| Category | Sample | *sul*1 | *sul*2 | *tet*(L) | *tet*(W) | *erm*(F) | *intI*1 |
| --- | --- | --- | --- | --- | --- | --- | --- |
| Potting Soil | POS1 | -4.3 | -1.9 | -4.6 | -4.1 | ND | ND |
|  | POS2 | ND | -3.6 | ND | -4.7 | ND | ND |
|  | POS3 | -3.1 | -2.8 | -4.1 | -3.9 | -2.5 | -3.0 |
|  | POS4 | -4.1 | -3.1 | -6.1 | -4.1 | ND | ND |
|  | POS5 | ND | ND | -5.1 | -4.0 | ND | ND |
|  | POS6 | -4.0 | -2.2 | -6.4 | -5.0 | -2.6 | ND |
|  | POS7 | ND | -4.2 | -4.9 | -4.5 | ND | ND |
|  | POS8 | -2.6 | -3.0 | -5.9 | -4.1 | -3.0 | -2.5 |
|  | POS9 | ND | -2.8 | -5.1 | -3.0 | -5.0 | ND |
|  | POS10 | -3.4 | -2.5 | -6.2 | -4.7 | -4.7 | -3.8 |
| Garden Soil | GS1 | -2.6 | -2.9 | -3.4 | -2.0 | -3.3 | -3.1 |
|  | GS2 | -2.8 | -2.3 | -4.7 | -4.1 | -3.3 | -3.4 |
|  | GS3 | -2.9 | -2.3 | -4.5 | -4.3 | -3.5 | -3.6 |
|  | GS4 | -4.1 | -3.6 | -5.9 | -4.4 | ND | -4.5 |
|  | GS5 | -2.8 | -1.1 | -6.0 | -4.0 | -3.1 | -3.5 |
|  | GS6 | -2.2 | -1.8 | -5.5 | -2.8 | -2.8 | -2.2 |
|  | GS7 | -3.2 | -2.3 | -6.8 | -4.5 | -3.6 | -3.5 |
| Fruit Amendment | FA1 | -2.8 | -2.0 | -2.2 | -0.4 | -2.9 | -2.6 |
|  | FA2 | -1.9 | -2.8 | -0.4 | -0.2 | -2.5 | -1.8 |
|  | FA3 | -2.4 | -2.6 | -2.0 | -1.1 | -2.0 | -2.0 |
|  | FA4 | -2.4 | -1.3 | -2.4 | -2.5 | -2.4 | -2.1 |
| Lawn Amendment | LA1 | -2.1 | -1.7 | -4.6 | -2.6 | -3.3 | -2.4 |
|  | LA2 | -3.0 | -2.9 | -5.1 | -4.3 | -2.5 | -3.5 |
|  | LA3 | -1.8 | -0.2 | -3.8 | -4.6 | -1.5 | -1.5 |
|  | LA4 | -3.1 | -2.2 | -3.5 | -3.5 | -2.4 | -2.8 |
| Manure | M1 | -2.8 | -1.6 | -5.3 | -3.1 | -2.6 | -3.0 |
|  | M2 | -2.7 | -2.1 | -3.2 | -0.9 | -2.2 | -3.0 |
|  | M3 | -2.8 | -2.6 | -3.2 | -2.7 | -5.1 | -3.3 |
|  | M4 | -2.2 | -1.1 | -4.9 | -3.5 | -1.4 | -2.2 |
|  | M5 | -2.1 | -1.6 | -2.7 | -2.5 | -1.6 | -2.2 |
|  | M6 | -2.4 | -2.7 | -1.8 | -3.4 | ND | -3.1 |
| Compost | C1 | -1.9 | -2.7 | -4.5 | -3.8 | -3.6 | -2.5 |
|  | C2 | -1.3 | -2.7 | -5.8 | -5.4 | -3.6 | -2.4 |
|  | C3 | -3.4 | -2.9 | -4.8 | -3.0 | ND | -3.4 |
| Recently Landscaped Soil | RL1 | -1.9 | -2.5 | -5.4 | -3.0 | -3.1 | -2.3 |
|  | RL2 | -3.6 | -2.7 | -5.1 | -3.7 | -6.1 | -3.9 |
|  | RL3 | -1.7 | -2.5 | -4.6 | -4.9 | -2.1 | -2.2 |
| Native Soil | NS1 | ND | ND | -5.8 | -4.5 | -4.8 | ND |
|  | NS2 | ND | ND | -4.8 | ND | ND | ND |
|  | NS3 | ND | ND | -4.9 | ND | ND | ND |
|  | NS4 | -4.2 | ND | -5.9 | -6.3 | ND | ND |
|  | NS5 | ND | -5.4 | ND | -6.1 | ND | ND |

**Table S7.** P-values from Wilcoxon hypothesis tests.

|  | OMRI vs Non-OMRI | | Garden Products vs Native Soils | | Manure Sourcing vs Non-manure Sourcing | |
| --- | --- | --- | --- | --- | --- | --- |
|  | Absolute | Relative | Absolute | Relative | Absolute | Relative |
| *sul*1 | 0.244 | 0.330 | 0.003 | 0.003 | 0.985 | 0.660 |
| *sul*2 | 0.560 | 0.375 | 0.001 | 0.001 | 0.721 | 0.612 |
| *erm*(F) | 0.155 | 0.098 | 0.011 | 0.013 | 0.527 | 0.150 |
| *tet*(L) | 0.036 | 0.053 | 0.078 | 0.115 | 0.002 | 0.002 |
| *tet*(W) | 0.166 | 0.143 | 0.002 | 0.001 | 0.008 | 0.008 |
| *int*I1 | 0.111 | 0.111 | 0.004 | 0.004 | 0.745 | 0.292 |

Echeverria-Palencia, C. M.; Thulsiraj, V.; Tran, N.; Ericksen, C. A.; Melendez, I.; Sanchez, M.

G.; Walpert, D.; Yuan, T.; Ficara, E.; Senthilkumar, N.; et al. Disparate Antibiotic Resistance Gene Quantities Revealed across 4 Major Cities in California: A Survey in Drinking Water, Air, and Soil at 24 Public Parks. *ACS Omega* **2017**, *2*, 2255–2263. https://doi.org/10.1021/acsomega.7b00118.

Ji, X.; Shen, Q.; Liu, F.; Ma, J.; Xu, G.; Wang, Y.; Wu, M. Antibiotic Resistance Gene Abundances Associated with Antibiotics and Heavy Metals in Animal Manures and Agricultural Soils Adjacent to Feedlots in Shanghai; China. *J. Hazard. Mater.* **2012**, *235*–*236*, 178–185. https://doi.org/10.1016/j.jhazmat.2012.07.040.

Knapp, C. W.; Dolfing, J.; Ehlert, P. A. I.; Graham, D. W. Evidence of Increasing Antibiotic Resistance Gene Abundances in Archived Soils since 1940. *Environ. Sci. Technol.* **2010**, *44* (2), 580–587. https://doi.org/10.1021/es901221x.

Luo, Y. I.; Mao, D.; Rysz, M. Trends in Antibiotic Resistance Genes Occurrence in the Haihe River , China. **2010**, *44* (19), 7220–7225.

Pei, R.; Kim, S.-C.; Carlson, K. H.; Pruden, A. Effect of River Landscape on the Sediment Concentrations of Antibiotics and Corresponding Antibiotic Resistance Genes (ARG). *Water Res.* **2006**, *40* (12), 2427–2435. https://doi.org/10.1016/j.watres.2006.04.017.

Zhou, T., Lu, J., Tong, Y., Li, S., & Wang, X. Distribution of antibiotic resistance genes in Bosten Lake, Xinjiang, China. **2014**, *Water Science and Technology*, *70*(5), 925–931. https://doi.org/10.2166/wst.2014.321
